# Systems Genetics of the Lateral Septal Nucleus in Mouse: Heritability, Genetic Control, and Covariation with Behavioral and Morphological Traits

**DOI:** 10.1371/journal.pone.0044236

**Published:** 2012-08-31

**Authors:** Alexander Talishinsky, Glenn D. Rosen

**Affiliations:** Department of Neurology, Beth Israel Deaconess Medical Center, Boston, Massachusetts, United States of America; University of Edinburgh, United Kingdom

## Abstract

The lateral septum has strong efferent projections to hypothalamic and midbrain regions, and has been associated with modulation of social behavior, anxiety, fear conditioning, memory-related behaviors, and the mesolimbic reward pathways. Understanding natural variation of lateral septal anatomy and function, as well as its genetic modulation, may provide important insights into individual differences in these evolutionarily important functions. Here we address these issues by using efficient and unbiased stereological probes to estimate the volume of the lateral septum in the BXD line of recombinant inbred mice. Lateral septum volume is a highly variable trait, with a 2.5-fold difference among animals. We find that this trait covaries with a number of behavioral and physiological phenotypes, many of which have already been associated with behaviors modulated by the lateral septum, such as spatial learning, anxiety, and reward-seeking. Heritability of lateral septal volume is moderate (*h* ^2^ = 0.52), and much of the heritable variation is caused by a locus on the distal portion of chromosome (Chr) 1. Composite interval analysis identified a secondary interval on Chr 2 that works additively with the Chr 1 locus to increase lateral septum volume. Using bioinformatic resources, we identified plausible candidate genes in both intervals that may influence the volume of this key nucleus, as well as associated behaviors.

## Introduction

The septum, a key component of the limbic system, is involved in the control of a wide variety of functions, including emotions, learning and memory, fear, reward-seeking behavior, and modulation of autonomic functions. Anatomically, the septum is divided into the lateral, medial, and posterior nuclei, with the former two nuclei comprising the largest portion of the septum. These nuclei have distinct patterns of connectivity and physiology and are each associated with modulation of different functions. The GABAergic and cholinergic septohippocampal projections from the medial septum, for example, play a role in the modulation of learning and memory [1], [2]. In contrast, the lateral septum has strong projections to hypothalamic and midbrain regions, and has been associated with the modulation of social behavior [3], anxiety as assessed by lateral plus maze or a forced swim test [4]–[6], the expression of contextual fear conditioning [7], and behaviors connected to the mesolimbic reward pathways, such as alcohol consumption and linking context with reward [7]–[9]. The lateral septum also receives substantial glutamatergic afferent projections from the hippocampus and amygdala, and has been associated with a number of memory-related behaviors [10]–[12].

Understanding the natural variation of lateral septal anatomy and linked functions may provide important insights into sources of individual differences in behavior and even disease risk. For example, Ophir and colleagues [3] found a correlation between the level of expression of various receptors (oxytocin and vasopressin) in the septum and social interactions in prairie voles. Males with high levels of the vasopressin and low levels of oxytocin expression were more likely to investigate females. However, nothing is known about the range of natural variation of lateral septal anatomy and how this variation might be linked to a potentially wide range of behaviors. Here we use of the large family of BXD recombinant inbred (RI) strains to systematically quantify the range of variation in the volume of the lateral septum and to define possible loci that contribute to both structural and behavioral differences among these isogenic lines of mice.

The BXD family is a collection of full inbred strains that were created by successive inbreeding of F2 progeny generated from matings of C57BL/6J (B6) and DBA/2J (D2) mice [13], [14]. These strains have been genotyped at over 3000 markers enabling quantitative trait loci (QTL) mapping with high precision using established tools [15]. The BXD family has now been systematically analyzed using advanced stereological methods for close to 15 years, and is part of a comprehensive genetic dissection of the CNS including deep data on the hippocampus [16]–[18], several thalamic nuclei [19], basolateral amygdala [20]–[22], striatum [23], [24], neocortex [25]–[27], olfactory bulb [28], and cerebellum [29]. Even more remarkably, there are deep gene expression data sets for many brain regions and thousands of behavioral phenotypes, which allows for detailed construction of networks of covariation [30]. In this experiment, we used stereological probes to accurately and efficiently estimate the volume of the lateral septum in the BXD RI set, and find that this trait covaries with a number of anatomic, behavioral, and physiological phenotypes, many of which are modulated by the lateral septum. We genetically map this trait to a QTL interval on the distal portion of chromosome (Chr) 1, which interacts with an interval on the proximal portion of Chr 2, and use bioinformatic resources to identify potential candidate genes and gene networks modulating the volume of this structure.

## Methods

### Subjects

All histologic data for this study were obtained from The Mouse Brain Library (MBL)–a physical and Internet resource that contains high-resolution images of histologically processed slides from over 3200 adult mouse brains (http://www.mbl.org). In this experiment, we used cresyl violet stained slides [31] from 362 of these mice (190 female, 172 male) belonging to 69 BXD RI strains and their parentals. The ages ranged from 34–694 days of age (mean ± SEM = 123.2±4.3). We used brains that were cut in either the coronal (N = 183) or horizontal (N = 179) plane [31].

### Stereology

**Figure 1:**
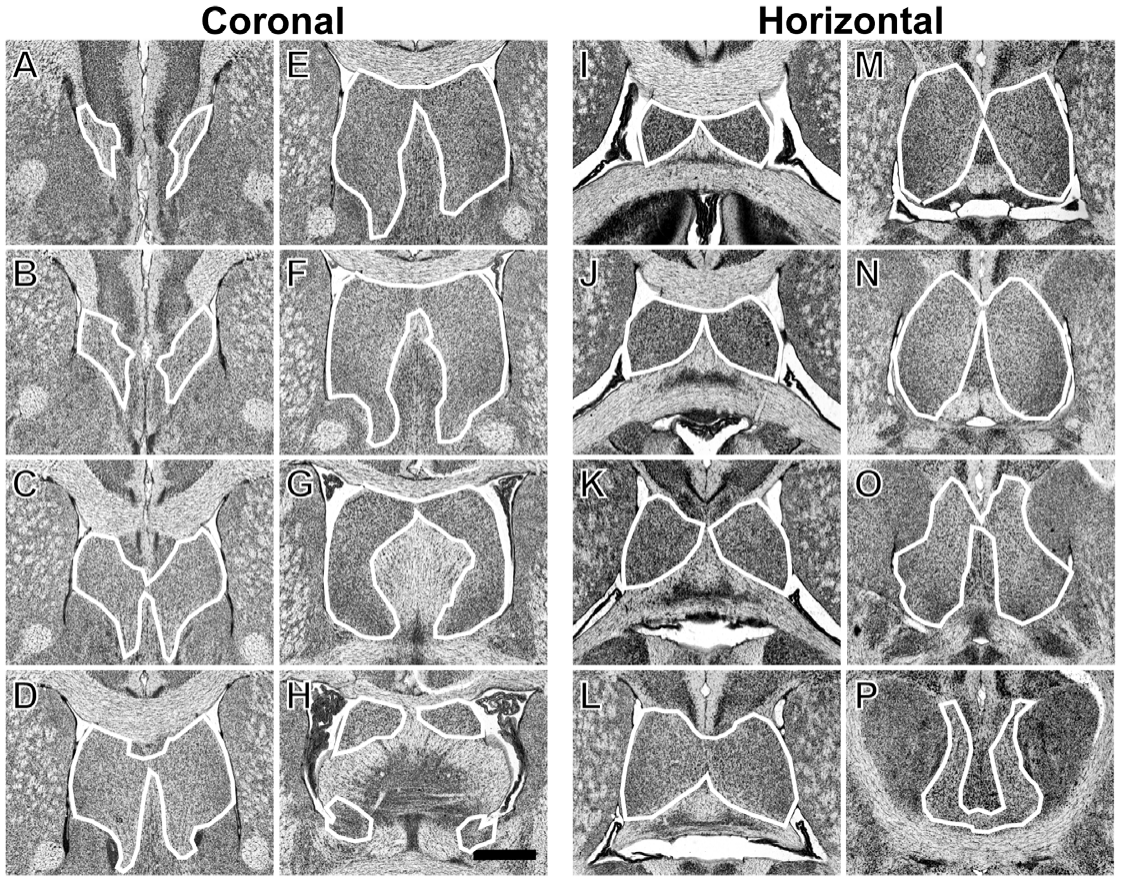
The parameters for the boundaries of the LS in coronal (A-H) and horizontal sections (I–P). In the coronal slides, LS begins just under the tenia tecta, forming a light, thin grainy tissue bordered by white matter (**A**). It then expands dorsally to form two large upturned triangular regions separated in the middle by the medial septum and bordered on the distal ends by the third ventricle (**B–D**). The ventral border of the LS is the line between the two Major Island of Calleja ventricles, or the line between the two nuclei of the anterior commissure olfactory limb if the former were not present in the coronal section. Moving caudally, the LS appears more dorsal and is bordered by the fornix, with a clear difference in the tissue between the lateral septum, the septofimbrial region (a darker region within the fornix included in the measurements), and the surrounding lighter regions of the fornix and darker regions of the stria terminalis (**E,F)**. In the caudal-most sections, the third ventricle separates the dorsal end of the LS as well as the fornix, and the remaining LS is easily distinguished from surrounding tissue (**G,H**). In the horizontal slides, the lateral septum is easily distinguished early on as two rounded bodes of dense grey matter between the curved arches of the cingulated gyrus (**I–K**). These two rounded bodies gradually grow in size and eventually meet just rostral to the darker matter of the medial septum and the less dense white matter of the fornix (**L**). Moving ventrally through the horizontal plane, the two rounded regions of the LS continue to grow outwards, and meet at their lateral anterior borders with the darker coarse grey matter of the nucleus accumbens (**N)**, which goes on to surround the LS on all but the caudal border, which is marked by the white arch of the anterior commissure (**P**). Dorsal to the anterior commissure (**O**), the caudal borders of the LS arc from the caudal end of the third ventricle to the rounded white matter of the fornix.

The volume of the lateral septum (LS) was estimated by one of us (AT) using a computer controlled microscope (Zeiss Axiophot, Zeiss, Melville, NY) and Stereo Investigator (MBFBiosciences, Williston, VT). We used the Cavalieri probe of Stereo Investigator to lay down a 250 × 250 µm grid, and we examined sections every 150 µm. Cavalieri’s method [32] was used to compute the estimate of LS volume. In rare cases of missing or damaged sections, a piece-wise parabolic estimation was used [33]. The borders of the lateral septum were those of Franklin and Paxinos [34] and are delineated in Fig. 1. Intra-observer reliability was assessed by blindly re-measuring 18 randomly chosen brains. The percentage difference between original and repeated volume estimations ranged from 0.18% to 12.73%, with a mean ± SEM of 3.91% ±0.8% deviation. A correlation coefficient between the two measurements was highly significant (r = 0.99) indicating that technical error at this level of the analysis contributes little to case variation or strain variation. A paired t-test confirmed that the difference between the first and second estimations was not significant (t <1, NS).

### Analysis

Data were analyzed using standard ANOVA and multiple regression techniques (JPM, SAS institute, Cary, NC, STATA/SE, Stata Corporation, College Station, TX). QTL analysis was performed using the WebQTL module of GeneNetwork (GN, http://www.genenetwork.org) [15]. This online resource includes all known morphometric, behavioral, and physiological data for the BXD strains, high density marker maps based on approximately 7000 fully informative markers distributed on all chromosome except Chr Y, and a database containing ∼9.3 million SNPS taken from dbSNP. WebQTL incorporates three common mapping methods: (1) simple interval mapping, (2) composite interval mapping, and (3) a scan for two-locus pair-wise interactions. To evaluate candidate genes we used the QTLMiner module of GN [35], which evaluates all genes in an interval against gene expression databases. There are no expression databases of the LS, so we employed mRNA expression databases from the hippocampus, amygdala, and hypothalamus– all of which have strong afferent and/or efferent connections to the LS. In addition, we used Gene Weaver [36] to determine overlap of positional gene candidates with gene expression in the Allen Brain Atlas [37]. To study covariates of LS volume we correlated these values with a database of over 3000 previously published and unpublished BXD traits in GN.

### Databases and On-line Resources

The Mouse Brain Library: <www.mbl.org>.

GeneNetwork: <www.genenetwork.org>.

QTLMiner: <http://www.genenetwork.org/webqtl/main.py?FormID=qtlminer>.

Allen Brain Atlas: <http://brain-map.org>.

Gene Weaver: <http://ontologicaldiscovery.org/>.

Hippocampus Consortium M430v2 (Jun06) PDNN Database: <http://www.genenetwork.org/dbdoc/HC_M2_0606_P.html>.

INIA Amygdala Cohort Affy MoGene 1.0 ST (Mar11) RMA: <http://www.genenetwork.org/dbdoc/INIA_AmgCoh_0311.html>.

INIA Hypothalamus Affy MoGene 1.0 ST (Nov10): <http://www.genenetwork.org/dbdoc/INIA_Hyp_RMA_1110.html>.

BXD Published Phenotypes Database: <http://www.genenetwork.org/dbdoc/BXDPublish.html>.

### Ethics Statement

This study was carried out in strict accordance with the recommendations in the Guide for the Care and Use of Laboratory Animals of the National Institutes of Health. The Mouse Brain Library was created under protocols approved by the Institutional Animal Care and Use Committees of Beth Israel Deaconess Medical Center and the University of Tennessee Health Science Center. The gene expression databases at GeneNetwork were created under protocols approved by the Institutional Animal Care and Use Committees of the University of Tennessee Health Science Center. All surgery was performed under anesthesia, and all efforts were made to minimize suffering.

## Results

### Lateral Septal Volume is Highly Variable

**Figure 2:**
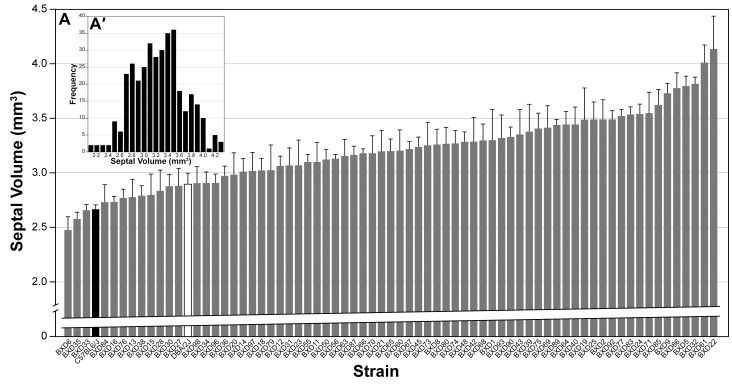
Histogram of mean (± SEM) LS volume (corrected for histological shrinkage) in 69 BXD strains (gray bars) and in the two parentals: DBA/2J (white bar) and C57BL/6J (black bar). Inset is frequency distribution of MSACC in all 362 subjects, illustrating a mostly normal distribution.

All estimates of LS volume are corrected on a case-by-case basis for shrinkage and should be considered close to the original size of these regions in well-fixed tissue. LS volume of individual mice ranges from 2.03–4.80 mm^3^ (mean ± SEM = 3.21±0.02 mm^3^), and is normally distributed (Fig. 2A′). Strain averages extend from a low of 2.48±0.12 mm^3^ in BXD6 to a high of 4.14±0.30 mm^3^ in BXD22 (Fig. 2A).

### Lateral Septal Volume is Highly Heritable

In order to assess heritability, we computed an ANOVA with *strain* as the independent measure and LS volume as the dependent measure. We found a significant effect of strain (*F*_70,291_ = 1.71, *P*<.005). The strain main effect accounts for 29% of the variance, which provides a reasonable upper bound on the fraction of variance that might be explained by additive and epistatic interactions. (Dominance effects cannot be measured using a set of RI strains because there are no heterozygous genotypes and phenotypes to measure). We also computed heritability (*h^2^*) by dividing the between strain variance by the total variance. LS volume is a highly heritable trait (*h^2^* = 0.52).

### Mapping Lateral Septal Volume

**Figure 3:**
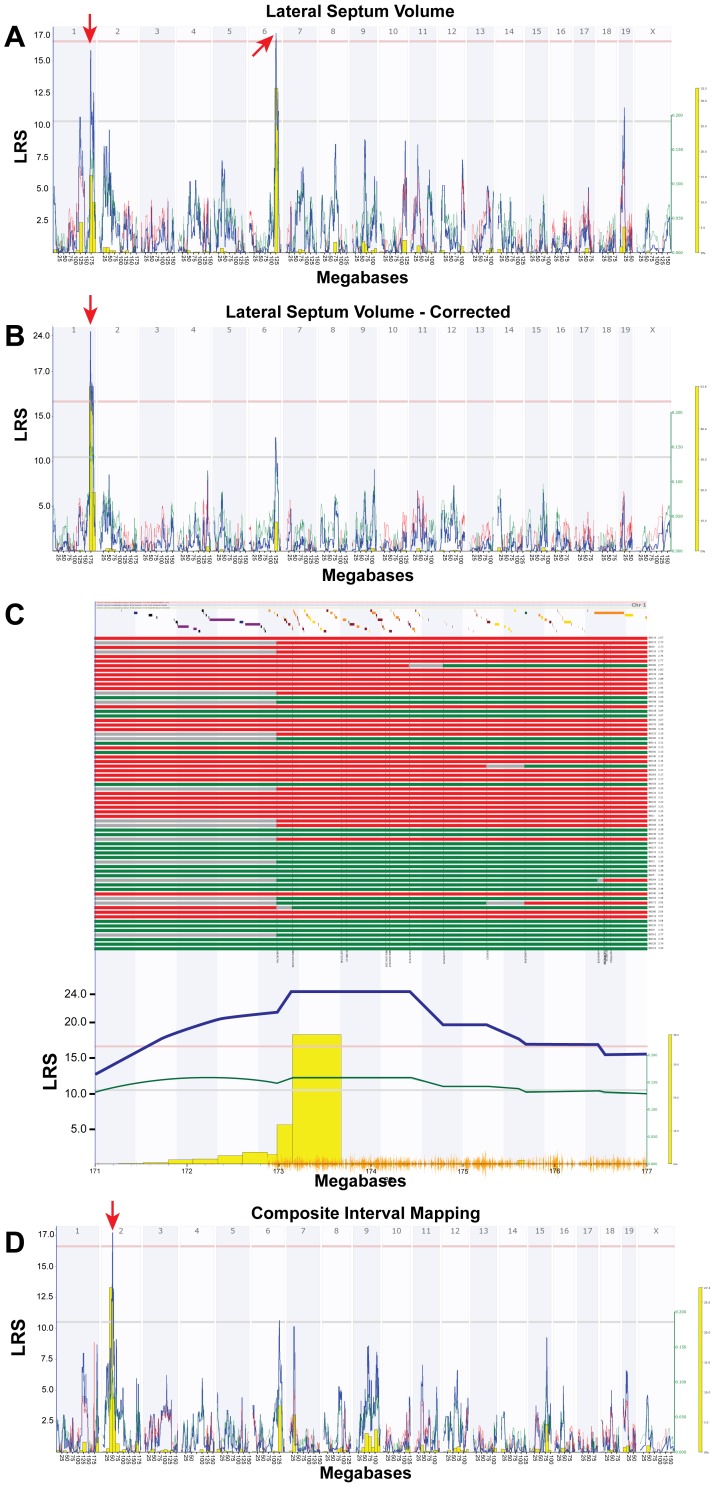
Mapping LS volume in BXD RI strains. A . Interval map of LS corrected for histological shrinkage across the entire genome. The x-axis represents the physical map of the genome; the y-axis and thick blue line provide the LRS of the association between the trait and the genotypes of markers. The two horizontal lines are the suggestive (blue) and significance (red) thresholds computed using 1000 permutations. There is a significant QTL mapping to the distal portion of Chr. 6 and a suggestive QTL mapping to the distal portion of Chr 1 (red arrow). **B**. Interval map of LS volume corrected for shrinkage with the effects of age, sex, epoch, and brain weight regressed out. The Chr 1 QTL seen in panel A is now significant (red arrow). **C**. Haplotype map of all 69 BXD strains on 6 Mb QTL interval on Chr1 (171–176 Mb). Red lines indicate C57BL/6J alleles (maternal), green lines indicate DBA/2J alleles (paternal), and gray lines are unknown. Strains are arranged from smallest to largest LS volume (top to bottom). D. Composite interval mapping indicates a QTL on the proximal portion of Chr 2 (45–55 Mb).

We mapped LS volume (corrected for histological shrinkage) and detected two loci (Fig. 3A) with closely matched likelihood ratio statistics (LRS), both of which were suggestive of linkage. The first locus on Chr 6 peaks between 124 and 132 Mb (LRS = 16.1). The second locus on Chr 1 peaks between 171 and 177 Mb (LRS = 15.8, Fig. 3A). Strains inheriting the D2 haplotype generally had larger LS volume (0.15 mm^3^ per allele at both QTLs) than those inheriting the B6 haplotype. In order to map variation related to LS volume rather than possible confounding covariates, we used multiple regression to remove all covariance associated with age, sex, plane of section, brain weight, and strain epoch (Taylor strains vs. UTHSC strains). We then computed the residuals for this regression, and mapped these residuals (Fig. 3B). The Chr 1 QTL was found to be significant (LRS = 24.4, *P*<.001), and the Chr 6 peak diminished, suggesting that the latter QTL mapped one of the regressed variables. We used the Haplotype Analyst function in GN to plot the pattern of inheritance in this interval (Fig. 3C). Strains inheriting D2 haplotypes generally had large lateral septum compared with those strains inheriting the B6 haplotype (0.15 mm^3^ per allele). Composite interval mapping controlling for the Chr 1 QTL revealed a significant QTL on Chr 2 between 45–55 Mb (LRS = 18.4, *P*<.05, Fig. 3D).

**Figure 4:**
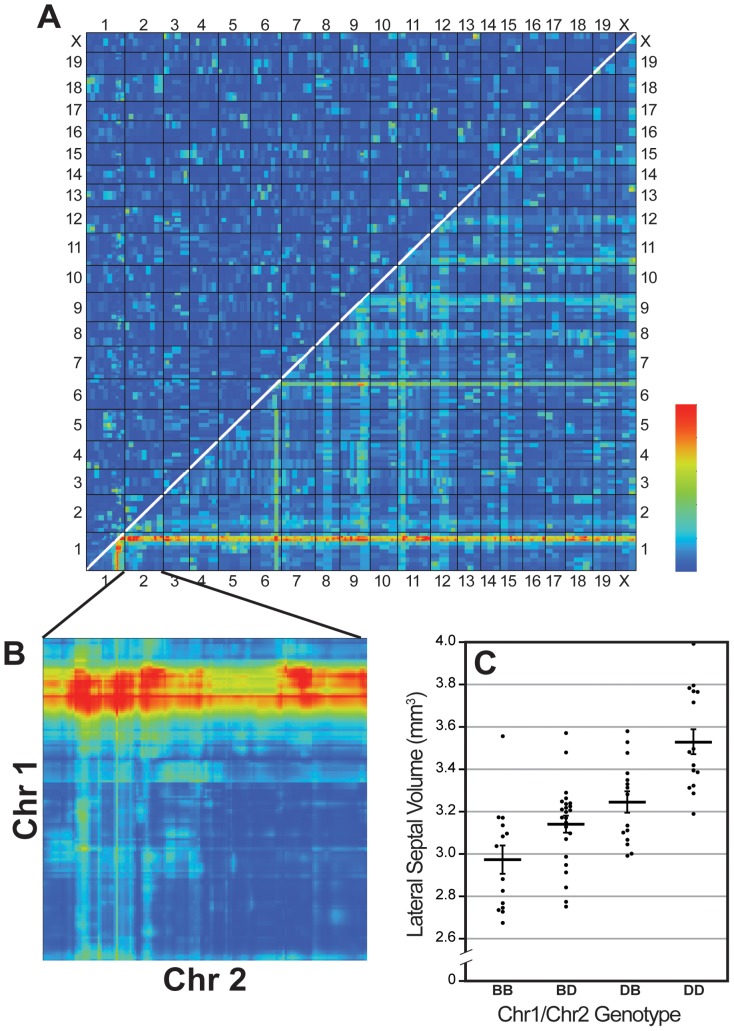
Pair-scan correlations demonstrate additive effect of QTLs on Chr1 and Chr 2. **A.** Pair-scan analysis of adjusted LS volume (corrected for shrinkage, age, sex, epoch, and brain weight) across the genome. The upper left half of the plot highlights any epistatic interactions, while the lower right half provides a summary of LRS of the full model, representing cumulative effects of linear and non-linear terms. **B**. A significant interaction between QTLs on Chr 1 and 2 (white circle) is shown. **C.** Scatterplot (with means ± SEM) illustrating the effect on adjusted LS volume of carrying either the parental (D2) or maternal (B6) or both alleles at the Chr 1 and Chr 2 intervals. Having D2 alleles at both intervals significantly increases adjusted striatal volume when compared to all other allelic combinations.

We also tested for pair-wise interactions using the pair scan module of GN (Fig. 4A). We found a significant interaction between the Chr 1 QTL interval and the Chr 2 interval that was identified using composite interval mapping (LRS = 43.1, *P*<.01, Fig. 4B). We performed an ANOVA with genotypes of markers in the Chr 1 and Chr 2 QTLs as the independent measures and the LS volume as the dependent measure. We found a significant main effect for both the Chr 1 QTL (*F*_1,64_ = 37.3, *P*<.001) and the Chr 2 QTL (*F*_1,64_ = 19.6, *P*<.01), but there was no significant interaction between the two QTL intervals (*F*_1,64_<1, NS). A one-way ANOVA revealed that those strains with D alleles at both intervals have significantly larger LS volume than any other combination of alleles (*F*_3,64_ = 18.1, *P*<.001; Fig. 4C), which supports an additive effect of these two loci.

### Candidate Gene Analysis

There were a total of 125 genes in the gene-rich Chr1 interval (171.00–177.0 Mb). Positional candidate genes were selected using a multistep process. To evaluate positional candidate genes, we used the QTLminer module of GN, which ranks genes by whether 1) the parent strains (B6 and D2) have non-synonymous SNPs or indels, 2) they are expressed in transcriptome databases, and 3) their expression is modulated by cis-eQTLs. GN does not have a gene expression database for the lateral septum, so we instead used mRNA expression databases from three regions with strong connections to the LS–hippocampus, amygdala, and hypothalamus. From the initial pool of all genes in the interval, QTLminer identified 50 genes that had non-synonymous SNPs or indels or polymorphic UTRs and were expressed in at least one of the three regions and whose expression was cis-modulated (cis-eQTL). To complement this analysis, we used Gene Weaver [36] to identify those genes within the interval that were also highly expressed in the LS of the Allen Brain Atlas. We identified 19 positional candidates, which are summarized in Table 1.

**Table 1:** Positional candidate genes in the Chr 1 interval.

Gene Symbol	Gene Description	nsSNPs(Exons)	ABA	eQTL
*Adamts4*	a disintegrin-like and metallopeptidase (reprolysin type) withthrombospondin type 1 motif, 4	5 (1,5,6)	No	Hi,A,Hy
*Atp1a2*	ATPase, Na+/K+ transporting, alpha 2 polypeptide	5 (13)	Yes	Hi,Hy
*B4galt3*	UDP-Gal:betaGlcNAc beta 1,4-galactosyltransferase, polypeptide 3	UTR	Yes	Hi,A,Hy
*Cadm3/Igsf4B*	cell adhesion molecule 3	UTR	Yes	Hi
*Darc*	DDB1 and CUL4 associated factor 8	9 (2)	Yes	A, Hy
*Fh1*	fumarate hydratase 1	1 (6)	Yes	-
*Fmn2*	formin 2	12 (1,3,5,6,11,13,14)*	Yes	Hi,A,Hy
*Igsf8*	immunoglobulin superfamily, member 8	2 (3,4,5,6)*	Yes	Hi,A
*Kcnj9/Girk3*	potassium inwardly-rectifying channel, subfamily J, member 9	UTR	Yes	Hy
*Kcnj10*	potassium inwardly-rectifying channel, subfamily J, member 10	1 (2)*	No	Hi,A,Hy
*Lin9*	lin-9 homolog (C. elegans)	2 (9,12)*	Yes	-
*Ndufs2*	NADH dehydrogenase (ubiquinone) Fe S protein 2	5 (1,2,6)*	Yes	Hi,A,Hy
*Pcp4l1*	Purkinje cell protein 4-like 1	UTR	Yes	Hi
*Pea15a*	phosphoprotein enriched in astrocytes 15A	UTR	Yes	Hi,Hy
*Pex19*	peroxisome biogenesis factor 19	1 (2)	No	A
*Pigm*	phosphatidylinositol glycan anchor biosynthesis, class M	1 (1)	No	A,Hy
*Rgs7*	regulator of G protein signaling 7	UTR	Yes	-
*Uhmk1*	U2AF homology motif (UHM) kinase 1	1 (1)	Yes	-
*Wdr42a/Dcaf8*	WD repeat domain 42A	UTR	Yes	A,Hy

Abbreviations:

nsSNPs – Non-synonymous single nucleotide polymorphisms or indels. Exons in parentheses, *indicates multiple transcripts.

ABA – Expression in the lateral septum of the Allen Brain Atlas.

eQTL - cis-eQTL expression in the Hippocampus (Hi), Amygdala (A) and/or Hypothalamus (Hy).

Pair-scan analysis revealed a significant interaction between the Chr 1 QTL and an interval on the proximal end of Chr 2 (45–55 Mb). We evaluated the genes on the Chr 2 interval using QTLMiner and Gene Weaver as described above. Of the 38 genes in the interval, we identified 12 that had SNPs and/or indels and/or polymorphisms in their UTRs, were expressed in the hippocampus, amygdala, and hypothalamus transcriptome databases, and had cis-eQTLs (Table 2). We examined the expression of these genes in each of the three databases for trans-QTLs in the Chr 1 interval, which might suggest genetic linkage between these genes, and found a modest trans eQTL at the Chr 1 locus for the expression of *Cacnb4* in the hippocampus (LRS = 11.5), and for *C1orf103* in the hippocampus and amygdala (LRS = 10.7 and 11.2, respectively). In a reciprocal manner, we examined expression of the genes in the Chr 1 QTL interval for trans eQTLs located in the Chr 2 interval and found a modest trans eQTL for *Uhmk1* (LRS = 7.9) in the Chr 2 interval.

**Table 2:** Positional candidate Genes in the Chr 2 Interval.

Gene Symbol	Gene Description	nsSNPs(Exons)	ABA	eQTL
*Arl6ip6*	ADP-ribosylation-like factor 6 interacting protein 6	1 (4)	No	–
*Acvr2*	Activin A receptor, type IC	–	No	Hi
*Arl5*	ADP-ribosylation factor-like 5; distal 3′ UTR	UTR	No	Hi
*Cacnb4*	Calcium channel, voltage-dependent, beta 4 subunit	UTR	No	A
*Fmnl2*	Formin-like 2	4 (1,6,7,8)	No	-
*Galnt13*	UDP-N-acetyl-alpha-D-galactosamine:polypeptide N-acetylgalactosaminyltransferase 13	UTR	Yes	Hi
*Lrif1*	ligand dependent nuclear receptor interacting factor 1	19 (17, 20, 25, 30)*	No	Hi
*Mbd5*	–	–	Yes	–
*Nmi*	N-myc (and STAT) interactor	4 (4, 5, 6, 7, 8, 9)*	No	Hi, A, Hy
*Rbm43*	RNA binding motif protein 43; 3′ UTR	UTR	No	Hi,A
*Rprm*	Reprimo, TP53 dependent G2 arrest mediator candidate	1 (1)	Yes	Hi
*Tnfalp6*	Tumor necrosis factor alpha induced protein 6	UTR	Yes	A, Hy

Abbreviations:

nsSNPs – Non-synonymous single nucleotide polymorphisms or indels. Exons in parentheses, *indicates multiple transcripts.

ABA – Expression in the lateral septum of the Allen Brain Atlas.

eQTL - cis-eQTL expression in the Hippocampus (Hi), Amygdala (A) and/or Hypothalamus (Hy).

**Figure 5:**
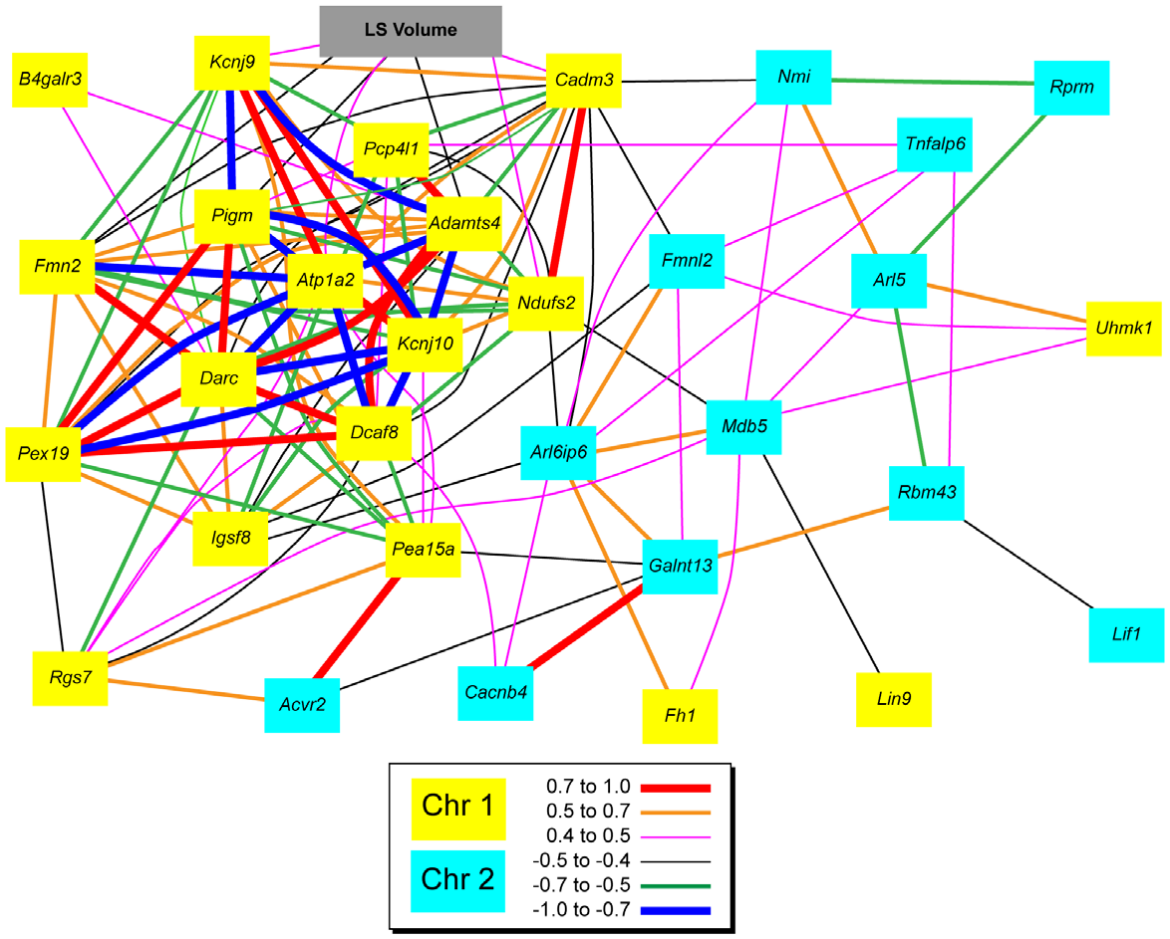
Covariation of LS volume (gray) with Chr 1 (yellow) and Chr 2 (blue) candidate gene expression in the hippocampus. Legend indicates the strength of the correlation between the genes.

Another method for exploring interactions between the Chr 1 and Chr 2 intervals is to examine covariation of candidate gene expression. We used the hippocampal expression database in GeneNetwork to identify candidate gene transcripts with high expression, and generated a network graph that illustrated their covariation (Fig. 5). This graph shows that *Ndufs2* in the Chr1 interval correlates positively with lateral septal volume and negatively with genes on the Chr 2 interval, both of which positively correlate with each other (*Arl6ip6* and *Mdb5*).

### Trait Correlation Analysis

**Figure 6:**
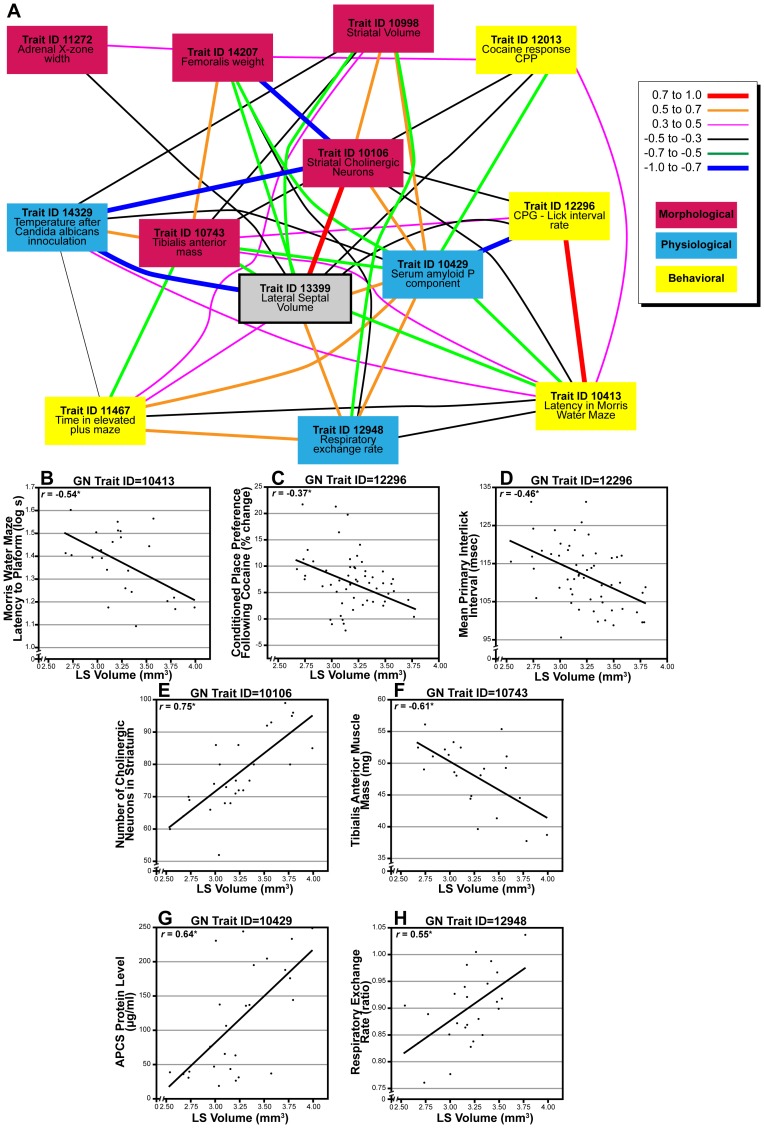
Covariation of LS volume with traits from the BXD Phenotype Database. **A**. Gene network diagram illustrating covariation among LS volume (gray) and a collection of morphological (red), physiological (blue), and behavioral (yellow) traits. Legend indicates the strength of the correlation between the genes. **B–H**. Scatterplots of LS volume correlated with behavioral (B-D), morphological (E,F) and physiological (G,H) traits.

LS volume was correlated with the BXD Published Phenotypes Database of GeneNetwork, which contains over 3,000 traits extracted from over 250 studies using BXD RI lines. Because of the large number of potential comparisons, we adjusted the alpha level for these correlations to α = .005 using criteria previously described [24]. Correlations of LS volume with behavioral, morphological, and physiological traits are summarized in Fig. 6A. This figure demonstrates not only the correlations with LS volume, but also the covariation among these traits.

Among behavioral traits, there were correlations between LS volume and traits that assess spatial learning, namely performance in the Morris water maze test. Specifically, strains with larger LS volumes were faster at finding the hidden platform than those with smaller LS volumes (*r* = 0.54, *N* = 25, *P*<.005, Fig. 6B, [38]). There were significant negative correlations of LS volume with a measure of susceptibility to substance use disorders, namely the percent increase of time spent in a chamber that contained cocaine in a previous trial (*r* = −0.37, *N* = 55, *P*<.005, Fig. 6C, [39]). Thus, strains with larger LS volumes are less susceptible to substance use disorders. Interestingly, LS volume correlated positively with an anxiety measure from this same study–time in the middle of an elevated plus maze (GN Trait ID 11467, *r* = 0.40, *N* = 55, *P*<.005). Finally, LS volume was found to negatively correlate (r = −0.46, N = 56, P<.005, Fig. 6D, [40]) with inter-lick intervals on a water bottle, which is a measure of central pattern generation [41].

Among morphological traits, LS volume correlated with the number of cholinergic neurons in the striatum (r = 0.75, N = 26, P<.005, Fig. 6E, [42]) and the volume of the striatum (*r* = 0.50, *N* = 51, *P*<.005, [24]), which is intriguing given the importance of the cholinergic system in mediating lateral septal function and reciprocal connectivity between the LS and striatum. We also found a negative correlation with the mass of the tibialis anterior muscle 6, indicating that a larger LS volume is associated with decreased muscle mass (r = −0.61, N = 22, P<.005, Fig. 6F, [43]). This is bolstered by the negative correlation with the mass of the femoralis muscle in unpublished data from G Brockmann and K Schughart (GN Trait ID 14207, *r* = −0.58, *P*<.005). The body weight of F1 hybrids from crosses of BXD mice to a transgenic C57BL/6J R6/2 model of Huntington’s disease correlated significantly with LS volume (*r* = 0.68, *N* = 15, *P*<.005, GN trait ID 12767 from R Paylor and colleagues). Significant correlations were found between LS volume and the width of the adrenal x-zone in males (r = −0.37, N = 57, P<.005, [44]) as well as the ratio retinal ganglion to lateral geniculate nucleus neurons (r = 0.50, N = 33, P<.005, [45]).

There is a highly positive correlation between LS volume and endogenous serum amyloid P levels–an acute phase reactant that increases in response to inflammatory stimuli (*r* = −0.61, *N* = 26, *P*<.005, Fig. 6G, [38]). If strains with larger volumes are have larger endogenous levels of serum amyloid P, then one might predict that these strains would also be more reactive to an inflammatory response. Unpublished data from L Toth (GN Trait ID 1439) suggests that strains with large LS volumes have a greater decrease in body temperature in response to inoculation with *Candida albicans* (*r* = −0.80, *N* = 12, *P*<.005). Finally, there was a significant positive correlation of LS volume with respiratory exchange rate of 13-week old males (r = 0.55, N = 25, P<.005, Fig. 6H, [46]).

## Discussion

In this experiment we have used classic stereological methods to estimate the volume of the lateral septum in a large panel of BXD recombinant inbred strains. We found a 2.4-fold difference in lateral septum volume among individuals and a 1.7-fold difference among strains. Other morphological, physiological, and behavioral BXD phenotypes known to be modulated by the lateral septum significantly covary with LS volume. Because the BXD RI lines are a genetic reference panel, we were able to map variation in LS volume to an interval on distal Chr 1. Using dense maps of polymorphisms between the two parental strains and large-scale transcriptome databases we have identified 19 positional candidate genes within this interval. We have also identified a locus on proximal Chr 2 that additively interacts with the Chr 1 QTL to increase LS volume.

### Covariation with Behavioral, Morphological, and Physiological Phenotypes

The lateral septum plays a modulatory role in the central nervous system by integrating cognitive, emotional and autonomous processes through a series of (mostly) reciprocal connections ranging from the telencephalon to the spinal cord [47], [48]. In the current experiment, we found that LS volume significantly correlated with a number of behavioral, morphological, and physiological phenotypes that reflect these functions.

It has long been known that lesions in the septal area–especially the anterior and lateral septal nuclei [49], [50]–lead to a “septal rage” syndrome, characterized by enhanced defensive behavior and increased aggression, which can be experimentally assessed by an increase in spontaneous mouse killing by rats. It has been argued that the lateral septum, among other regions, normally plays an inhibitory role in predatory mouse killing, and may modulate general anxiety responses, such as predation, aggression, and social defensiveness [51]. Previous research has demonstrated that the LS (in conjunction with hippocampal inputs) plays an important role in modulating anxiety as measured in a plus maze [52], [53]. Evidence from the current experiment suggests that an increase in LS volume is associated with a decrease in anxiety as assessed by time in the middle of an elevated plus maze [39].

Because of its connections to mesolimbic reward pathways [4], [54], the septum has been associated with susceptibility to substance use disorders. The LS, in particular, has been shown to regulate ethanol consumption [8], [55]. Moreover, the LS showed greater neural activity following alcohol withdrawal [56]. This particular study used the parent strains of the BXD RI line (B6 and D2), which are known to differ in sensitivity to alcohol and to its withdrawal [57]. D2 mice showed an increased neural response (as measured by c-fos activity) to withdrawal compared to B6 mice. In the current study, increased LS volume was associated with a decrease in time spent in a compartment where cocaine had been previously available, as well as a decrease in average daily ethanol consumption [39], suggesting that increased lateral septal volume may lead to decreased substance abuse susceptibility. This association makes sense in light of the previously drawn association between lateral septal volume and decreased anxiety; It has been found that social anxiety can increase the acute rewarding properties of cocaine [58], so it is possible that the lateral septum’s role in modulating anxiety also affects substance use behaviors.

On the anatomical level, LS volume correlated significantly with the number of cholinergic neurons in the striatum [42], as well as to the volume of the striatum [24]. There is strong cholinergic input to both the striatum and LS, and there is evidence that the LS modulates anxiety in parallel with the striatum through a system of dorsal raphe serotonergic neurons [59], [60]. Interestingly, both LS volume and the number of striatal cholinergic neurons have strong correlations to measures of infectious disease susceptibility. In one instance, we found highly positive correlation with endogenous serum amyloid P levels [38]. Because higher levels of endogenous serum P are indicative of increased sensitivity to infection, it is perhaps not surprising that we also found that strains with larger LS volume had a greater decrease in body temperature after inoculation with *Candida albicans*, a fungus that is a causal agent of opportunistic oral and genital infections in humans (L Toth, GN Trait ID 14329).

Taken together, many of the traits that covary with LS volume would be predicted by the known functions of the lateral septum. In the behavioral realm, the septum is known to mediate anxiety and reward-response behaviors. Here we find that an increase in LS volume is associated with a general decrease in anxiety and in susceptibility to substance use disorders. From the anatomical perspective, one might expect correlations with the striatum because of their strong anatomical connections. On the other hand, LS volume covaried with some surprising traits. For example, the relationship of LS volume to infectious disease susceptibility was novel, as there is no literature directly linking these traits. Similarly, the negative correlation with a measure of central pattern generation, which is modulated by cranial nerves [61] is surprising. These results will need to be replicated before further investigation of their relationship to LS volume is warranted.

### Candidate Genes

The gene-rich Chr 1 QTL region has been previously identified as a hotspot that modulates a number of behavioral phenotypes and gene expression [62]. A number of the candidate genes from this region are associated with phenotypes linked to LS function. *Atp1a2* knockouts have enhanced emotionality on open-field and elevated-plus maze tests [63]. *Kcnj10* is associated with ethanol consumption [64] as well as increased seizure susceptibility in mice and humans [65]–[67]. *Kcnj9*/*Girk3*, a G protein-activated inward rectifier potassium channel gene, is involved in analgesic regulation, drug response, and hippocampal neurite growth [68]–[70], and has been identified as a candidate gene related to a seizure susceptibility QTL [66], [67]. Interestingly, *Rgs7* facilitates functional coupling of G protein signaling proteins and regulator proteins to GIRK receptors in neurons [71]. In the striatum, sensitivity of motor stimulation to cocaine is dependent on *Rgs7* [72]. In addition to their effects on behavioral phenotypes related to the lateral septum, *Kcnj10*, *Kcnj9*, and *Rgs7* all play the structural role of modulating G-protein inwardly rectifying potassium channels.

Four of the selected candidate genes are similarly involved in CNS structure as well as growth. *Ndufs2* mutations are part of complex I deficiency disorders such as Leigh’s disease and mitochondrial diseases that include encephalopathies [73]–[75], and this gene was identified as a possible modulator of adult neurogenesis in the hippocampus [76]. Neurite extension is promoted by *Adamts4* [77], and *Fmn2* is largely expressed in the olfactory bulb, cortex, thalamus, hypothalamus, hippocampus and cerebellum during development [78], and has been identified as a strong modulator of a number of genes in this interval that affect RNA metabolism and protein synthesis [62]. *Igfs8* regulates early neural development [79] as does *Pcp4l1*, which is also expressed in adulthood in the septal nuclei [80].

Seizure susceptibility has been linked to mutations in four selected candidate genes; *Pex19* [81], *Kcnj10* [82], *Pigm* [83], and *Cabc1* [84]. There is a reduction in LS area as well as the number of GABAergic neurons in rats with pilocarpine-induced status epilepticus [85]. Interestingly, the onset of cortical deactivation and subsequent behavioral change during seizures has been associated with the spread of seizure activity to subcortical structures necessary for cortical function, including the LS [86]. These and other studies are suggestive of a modulatory role for the LS during seizures [87]. There are no behavioral associations with *Cadm3*, but it is a cell adhesion molecule expressed during nervous system development [88]. *Igfs8* regulates early neural development [79]. *Pcp4l1* is also expressed during early brain development and is expressed in adulthood in the septal nuclei [80].

Pair-scan analysis revealed a significant interaction between the Chr 1 QTL and an interval on the proximal end of Chr 2 (45–55 Mb), an interval that was also identified using composite interval mapping. These two loci act additively to increase LS volume, as strains with D2 alleles at both intervals had significantly larger volumes than those with any other combination of inheritance. Within the Chr 2 interval, we identified 12 genes as positional candidates.

We probed bioinformatic resources for evidence of interaction among the genes on the Chr 1 and Chr 2 intervals. Using the hippocampal mRNA expression database we found that *Cacnb4* had a modest trans-eQTL in the Chr 1 interval. Our gene expression co-variation network (Fig. 5) indicates that this gene co-varies with the Chr 1 positional candidate *Dcaf8*, as well as the Chr 2 candidates *Arl6ip6* and *Galnt13*. *Cacnb4* has been identified as the gene responsible for the lethargic (lh) mouse phenotype, which includes ataxia and absence epilepsy [89]. Our gene expression co-variation network (Fig. 5) indicates that this gene co-varies with the Chr 1 positional candidate *Dcaf8*, as well as the Chr 2 candidates *Arl6ip6* and *Galnt13*.The function of *Galnt13* in the brain is not known, nor is function of *Arl6ip6*, but a recent GWAS of susceptibility to ischemic strokes identified SNPs of this gene (along with *Fmnl2*) as potential contributors to this phenotype [90]. In a reciprocal manner, we found a modest trans-eQTL for the Chr 1 positional candidate *Uhmk1* directed at the Chr 2 interval. This gene covaries with a number of Chr 2 positional candidates, including *Arl5*, *Mbd5*, and *Fmnl2*. *Mbd5* has recently been identified as the causal locus of the 2q23.1 microdeletion syndrome in humans, the symptoms of which are intellectual disability, epilepsy, and autism spectrum disorders [91]–[93]. *Mbd5* is highly expressed in the hippocampus and moderately expressed in the lateral septum (Allen Brain Atlas, <brain-map.org>). *Fmnl2* is an analog of the Chr1 candidate for LS volume regulation, *Fmn2*. Like its Chr 1 analog, *Fmnl2* also plays a role in cell development and motility [94], and is highly expressed in human [95] and mouse brain (GeneNetwork, <genenetwork.org>).

Covariation analysis of LS volume with hippocampal gene expression identified *Ndufs2* as a potential link between Chr 1 positional candidates and those of Chr 2 (Fig. 5). LS volume correlates with *Ndufs2* expression in the hippocampus, which negatively correlates with both *Arl6ip6* and *Mbd5* expression, which in turn highly correlate with each other. *Arl6ip6* and *Mbd5* are central nodes in the covariation graph, correlating highly with nearly all of the other positional candidates in the Chr 2 interval, which suggests that they might play a major modulatory role of these Chr 2 genes. In addition, both *Arl6ip6* and *Mbd5* covary with the Chr 1 positional candidate *Fh1*. LS Volume also correlates with *Cadm3* in the Chr 1 interval, which in turn negatively correlates with *Nmi* and *Fmnl2* in the Chr 2 interval. *Nmi* is expressed in the brain [96], but it functions primarily as an oncogene.

Because there is no BXD gene expression database for the lateral septum, we queried gene expression databases from closely connected regions of the brain: hippocampus, amygdala, and hypothalamus. The Hippocampus Consortium M430v2 (Jun06) PDNN database in gene network [97], is an extensive database that contains steady state mRNA from 99 adult strains, including 67 BXD strains (all but 4 of which overlap with the current study). In comparison, the INIA Amygdala Cohort Affy MoGene 1.0 ST (Mar11) RMA and INIA Hypothalamus Affy MoGene 1.0 ST (Nov10) mRNA expression databases are less extensive, with 38 BXD strains common to the current study. We therefore attach greater weight to the data from the hippocampus database, although we approach data derived from these databases with some caution.

### Conclusions

The extensive genetic, morphologic, physiologic, and behavioral data that has been amassed using the BXD family of strains allows for powerful systems level dissection of a variety of traits. Here, we estimated the volume of the lateral septum, a limbic region known to modulate sociability, anxiety, fear conditioning, addiction, as well as a number of memory-related behaviors. We have demonstrated LS volume is a highly variable and highly heritable trait. Natural variation in the volume of LS volume covaries with a wide variety of behaviors. Specifically, increased LS volume is associated with a decreased anxiety and susceptibility to substance use disorders and better performance on a spatial memory task. We mapped a QTL to an interval on distal Chr1 that interacts additively with an interval on proximal Chr 2 to modulate LS volume. Further refinement of these gene-dense QTL intervals could be facilitated by selective phenotyping of inbred collaborative cross mice [98].
